# BK Polyomavirus in Renal Transplantation: Virological Notes for Monitoring and Diagnosis

**DOI:** 10.3390/biom16010052

**Published:** 2025-12-29

**Authors:** Cristina Costa, Francesca Sidoti, Alessandro Bondi, Antonio Curtoni

**Affiliations:** 1 University Hospital Città della Salute e della Scienza di Torino, 10126 Turin, Italy; francesca.sidoti@unito.it (F.S.); alessandro.bondi@unito.it (A.B.); antonio.curtoni@unito.it (A.C.); 2 Department of Public Health and Pediatric Sciences, University of Turin, 10126 Turin, Italy

**Keywords:** polyomavirus BKV, Polyomavirus-associated nephropathy, renal transplantation

## Abstract

Polyomavirus-associated nephropathy was first reported over 50 years ago. However, it still represents a cause of renal injury in kidney transplant recipients, particularly in the first two years post-transplantation, with occurrence rates of 1–10%. The role played by immunosuppression in viral reactivation is well acknowledged, and the modulation of its level is the main strategy for clinical management. Viral and immunological evaluation are fundamental for optimizing its diagnostic and therapeutic pathway. In this review, the main features of BK polyomavirus and associated nephropathy in renal transplant patients are addressed and discussed from a virological point of view; the role of BK polyomavirus in hematopoietic stem cell transplantation and other solid-organ transplant patients is also briefly reported.

## 1. *Polyomaviridae* Family

At present, fifteen human polyomaviruses have been described (Table 1) [1,2,3,4,5,6,7,8,9,10,11,12,13,14,15,16], in addition to two primate viruses also found in humans, i.e., SV-40 described in the 1960s and B-lymphotropic polyomavirus (PyV) in 2010. The first member of this family, i.e., murine polyomavirus (MPyV), was described in 1953 by Ludwig Gross and given the name “polyoma” (from the Greek: poly = many; oma = tumor) because of its ability to induce tumor development; in particular, it was observed to cause adenocarcinoma of the parotid glands in newborn mice. In 1971, the human BK polyomavirus was first reported in urine from a kidney transplant patient with ureteral stenosis [2], while JC was first isolated in the postmortem brain tissue of a patient with progressive multifocal leukoencephalopathy [3]; the two viruses were named after the initials of the two patients. Nevertheless, in the 1980s up to the late 1990s, polyomavirus-associated nephropathy was still neglected as a specific disease in the context of kidney transplantation, as at that time the main focus was devoted to managing and preventing graft rejection. It was only at the end of the last century that BK virus in kidney transplantation was considered a routine part of patient evaluation, although always in the absence of specific indications for the treatment. From 2007 to now, other polyomaviruses have been isolated, mainly in subjects with immunosuppressive conditions, although in most cases with no specific disease association [4,5,6,7,8,9,10,11,12,13,14,15]. Among these viruses, eight have been found on healthy skin and are actually considered part of the human cutaneous virome. Indeed, some of these viruses are not linked to specific diseases and could be an expression of transient infection, environmental exposure, or viral contamination.

The most pathologically important cutaneous polyomavirus, MCPyV, causes the majority of Merkel cell carcinomas: rare but highly aggressive malignant neoplasms occurring in both immunocompromised and immunocompetent individuals [6]. Moreover, primary infection with trichodysplasia spinulosa-associated polyomavirus (TSPyV) is linked to the very rare occurrence of this disease in immunocompromised subjects [8]. Research on blood donors has demonstrated the ubiquitous occurrence of polyomaviruses in the human population: antibodies against at least four polyomaviruses were reported in all individuals, with a seroprevalence rate ranging from 60% to 100% [17].

## 2. BK Polyomavirus and Polyomavirus-Associated Nephropathy

When the polyomavirus BKV was first isolated [2], its pathogenic role remained elusive, and it was considered an orphan virus for many years afterwards.

In 1978 and subsequent years, the main features of nephropathy in kidney transplantation were described, including the detection of urine decoy cells, the presence of viral inclusions in uroepithelial cells in graft biopsies, the challenging differential diagnosis with acute rejection, and the role of immunosuppression in the development of renal damage [18,19,20,21]. Nevertheless, polyomavirus-associated nephropathy was not yet recognized as a specific entity, and no specific diagnostic or therapeutic approach was considered. In 1995, Purighalla and colleagues first described a case of polyomavirus-associated nephropathy (PVAN), thus leading to its recognition as a definite disease entity [22]. Subsequently, several reports from many transplant centers worldwide with increasing prevalence rates have been published.

Although the epidemiology of PVAN may vary in different transplant centers, its incidence has been reported in 1–10% of kidney transplant recipients, mainly in the first two years following transplantation, and potentially leading to graft loss and return to hemodialysis in 30–80% of cases within 6–60 months [23,24]. Therefore, PVAN is associated with a significant impact on quality of life and the outcome of transplant procedures, not to mention the economic burden on national health care systems. Considering PVAN’s etiology, BKV accounts for most cases, whereas JCV has rarely been associated with nephropathy (<1–3%), either alone or in association with BKV [25,26,27,28,29,30,31,32].

BKV belongs to the genus Betapolyomavirus of the *Polyomaviridae* family and is a small, non-enveloped, icosahedral, and double-stranded virus, with a diameter of about 40–44 nm and a genome consisting of approximately 5000 base pairs. The genome is constituted by three regions: the early and late coding regions and the non-coding control region (NCCR). The early coding region encodes regulatory proteins involved in viral replication and transcription: large T antigen (AgT) and small t antigen (Agt); these genes are transcribed before viral DNA replication. Both AgT and Agt play a key role in malignant transformation; in fact, BKV and JCV are considered to be possible human carcinogens (i.e., listed in Group 2B by the IARC, https://monographs.iarc.who.int/list-of-classifications, accessed on 23 December 2025), although their causative role in human carcinogenesis remains controversial. The late coding region encodes the structural proteins VP1, VP2, and VP3 and the non-structural agnoprotein. Whereas the early and late coding regions are highly conserved, the NCCR sequence displays high variability with the possible emergence of mutant strains that may determine changes in cell tropism, viral fitness, potential for replication, features of pathogenicity, and virulence. The VP1 protein also exhibits high genetic variability. Based on single-nucleotide polymorphisms in VP1 and NCCR, BKV can be divided into four main genotypes: I–IV. Type I is the prevalent circulating strain and accounts for the majority of BKV-related disease. Types I and IV can be further divided into four (Ia, Ib-1, Ib-2, and Ic) and six (IVa-1, IVa-2, IVb-1, IVb-2, IVc-1, and IVc-2) subtypes. Globally, type I accounts for approximately 80% of viral isolates and type IV for approximately 15%, whereas types II and III are poorly represented. Molecular variants (genotypes and subtypes) present differences in terms of geographical distribution; for instance, subtype Ia is the most frequent in Africa, Ib1 in Southeast Asia, and Ib-2 in Europe, whereas Ic is the most common in Northeast Asia. The genotypes may present different biological behavior, which also determines differences from a clinical point of view; for example, type I strains have higher growth in human uroepithelial cells [33].

BKV is ubiquitously distributed in the population with a seroprevalence rate of up to 90% by the age of four [34]. Primary infection usually occurs in early childhood, probably by mucosal contact in the oral and respiratory tracts, and is often asymptomatic or presents only mild upper airway manifestations. Subsequently, BKV can establish a lifelong latent infection in uroepithelial cells as the most relevant latency site, as well as more rarely in other cell types (e.g., endothelial cells, fibroblasts, B cells, and brain) [35]. Immunocompetent individuals usually exhibit no significant clinical symptoms throughout their lives, with the possible exception of transient viruria. In immunocompromised patients, such as solid-organ transplant and hematopoietic stem cell transplant recipients and AIDS patients, BKV may reactivate due to impairment of the cell-mediated immune response. In particular, in kidney transplant patients, reactivation manifests as sequential occurrences of viruria and viremia [36], with tubular epithelial cells being the main target and leading to the development of tubulointerstitial nephritis. The incidence of BK viruria and viremia in kidney transplantation is reported to be approximately 30% and 12%, respectively [1], with viremia usually occurring within 2 to 6 weeks after the onset of viruria, with approximately half of viremic patients eventually progressing toward PVAN. Considering the role played by immunity in the onset of PVAN, most cases usually occur in the first two years following renal transplantation. Nephropathy of the native kidney of other solid-organ transplant recipients has also been described, mainly as case reports (see Section 4); in stem cell transplant recipients, BKV reactivation may be associated with hemorrhagic cystitis or hematuria with interstitial nephritis (see Section 5). The *condicio sine qua non* in the onset of viral reactivation and consequent disease is the net state of immunosuppression.

Although there are no vaccines or antiviral agents established for the treatment of PVAN, clinical management may benefit from significant advances and ameliorations obtained in recent years in terms of monitoring strategies, diagnostics, and therapeutic approaches.

## 3. Strategies for Diagnosing, Monitoring, and Managing Polyomavirus-Associated Nephropathy

Recently, the American Society of Transplantation Infectious Diseases Community of Practice has published guidelines for BKV in solid-organ transplantation [37], and the Transplantation Society International BK Polyomavirus Consensus Group has updated its recommendations by publishing the Second International Consensus Guidelines on the Management of BK Polyomavirus in Kidney Transplantation in 2024 [38].

According to these documents, particular attention is given to risk factors associated with PVAN onset, usually considered in terms of donor, recipient, and transplantation determinants, when considering the strategies for its monitoring and management. Among donor-related factors, the occurrence of viruria (i.e., urinary shedding of BKV), high anti-BKV-VP1 antibody levels, and certain BKV genotypes, as well as mismatches between donor and recipient genotypes, are considered the main determinants. Recipient-related risk factors for BKV viremia include older age and male gender, seronegativity for anti-BKV-VP1 antibodies, previous kidney transplantation, low recipient neutralizing antibody levels against the donor BKV serotype, and the absence of certain potentially protective HLA types (such as A2, A24, B7, B8, B13, B44, B51, Cw7, and DR15). In pediatric transplantation, younger recipient age and obstructive uropathy as the primary renal disease also represent risk determinants. Considering factors related to transplantation, a higher risk of viremia seems to be associated with the administration of specific immunosuppressive agents, e.g., tacrolimus in comparison to cyclosporine, T cell-depleting agents, high corticosteroid use, acute rejection events, AB0-incompatible transplants, and ureteric stents. Eder and colleagues [39] recently performed an extensive meta-analysis of 165 publications, including prospective and retrospective studies, encompassing more than 197,000 patients. The authors evaluated modifiable risk factors in relation to main endpoints, including biopsy-proven and presumptive PVAN, occurrence of viremia, and infection requiring treatment, and concluded that no single independent risk factor for all endpoints was identifiable, thus underlining the complexity in pathogenesis and in predicting BKV-associated impacts and complications in renal transplant recipients. Overall, it is likely that the overall level of immunosuppression, rather than a single drug or determinant, leads to the onset of nephropathy.

According to the above-mentioned guidelines, a possible diagnosis of PVAN is made in the presence of a urine viral load > 10 million genome copies/mL; a probable diagnosis in the presence of BKV DNaemia > 1000 genome copies/mL for at least two weeks; and a presumptive diagnosis for increasing viremia > 100,000 [37,38]. Biopsy-proven BKV nephropathy is diagnosed on the basis of detecting suggestive cytopathic effects plus immunohistochemistry and a specific diagnostic test for BKV to differentiate it from JCV [40,41,42,43].

As the impairment in immune response is the *condicio sine qua non* for the development of PVAN, and given the fact that there is no effective antiviral agent to prevent or treat BKV replication, the milestone for the clinical management of sustained BKV-DNAemia or biopsy-proven nephropathy in kidney transplant patients relies on the modulation of immune suppression with the aim of obtaining sufficient BKV-specific immune control and avoiding rejection episodes. Taking this into account, early evidence of viral reactivation is fundamental for a “preemptive” reduction in immune suppression, and monitoring for BKV should be focused on viral replication and, potentially, cellular immune response evaluation.

The knowledge of biological behavior and the clinical course of viral reactivation and nephropathy are the key points for establishing a monitoring strategy. Viral reactivation usually starts with the appearance of persistent asymptomatic viruria (>10^6^ copies/mL) or the occurrence of decoy cells in urine. These are virally infected uroepithelial cells observed in the urine cytology of kidney transplant recipients; decoy cells are characteristically identified by their enlarged nuclei, intranuclear inclusions, and other morphological changes indicative of viral infection. It should be noted that, although decoy cells can be used to screen for BKV infection, they are not always specific for it and may be present in other conditions, thus exhibiting a low sensitivity overall [42,43]. Viruria is typically followed by viremia within two to six weeks. Persistent viremia (>10^4–5^ copies/mL for at least three consecutive weeks) is usually indicative of uncontrolled viral replication, thus leading to tissue damage. Viruria and viremia usually precede the increase in serum creatinine. Despite the availability of these surrogate markers of viral replication, the definitive diagnosis of PVAN is made by histopathology. The sensitivity of histological diagnosis is directly proportional to the time from the occurrence of viremia and is related to viral load (in both urine and plasma). A negative histology is relatively frequent in the initial stages of PVAN [43], as well as being potentially challenging in consideration of the multifocal nature of the disease, with possible false negative results. Although not routinely performed, quantitative evaluation of BKV DNA on kidney biopsy by molecular assay could also be performed, with high viral loads (>10^4^ genome equivalents/cell) being associated with confirmed PVAN at histopathological evaluation [44,45]. Options for management are related to the stage of disease progression. In patients with viruria and viremia, a preemptive reduction in immune suppression before the occurrence of tissue damage may lead to resolution in 85–90% of cases and maintenance of renal function. Subsequently, in cases of late diagnosis with already established tissue damage, resolution is less probable and the rate of early loss of the graft rises from less than 10% up to 30%. In end-stage PVAN, treatment is usually late and inefficacious with progressive obliteration of renal tubula (the primary target of BKV infection) and a progressive decrease in viruria and viremia [46].

According to the consensus recommendations [38], monitoring viral replication allows for the identification of patients at risk, with a negative predictive value of 100% (i.e., no replication = no PVAN) and evaluation of a temporal profile of replication. Overall, this is crucial for timely management and subsequent monitoring of the response to treatment (i.e., modulation of immune suppression), as supported by several studies [45,46,47]. Monitoring for BKV replication is regularly recommended in renal transplant recipients and consists of the quantification of BKV-DNA on plasma specimens monthly up to 9 months post-transplantation and subsequently every 3 months for up to 2 years or in the occurrence of events such as graft dysfunction or renal biopsy (performed for surveillance or upon clinical indication: suspected rejection or nephropathy). In cases of persistent BKV-DNA in plasma (>10^4^ copies/mL), viremia should be re-evaluated within 2–3 weeks, and monitoring should be continued at 2–4-week intervals (Table 2). In cases of combined renal and other solid-organ transplantation, monitoring should be performed every three months up to three years post-transplantation. In other solid-organ transplantation, routine evaluation of BKV is not indicated, but it could be considered upon the occurrence of renal dysfunction; in this context, if a renal biopsy is taken, PVAN should be evaluated.

As regards the potential usefulness of a kidney biopsy, definitive diagnosis is performed by histological analysis. Studies have not evidenced a relation between histopathological staging and the level of viral replication (BKV DNAemia); for instance, we previously reported a wide range of viremia (from 10^4^ to 10^6^ copies/mL) in biopsy-proven nephropathy [45,48]. According to our center’s practice, monitoring for PVAN is actually undertaken by evaluation of BKV DNA on plasma specimens as recommended by the international guidelines. In the presence of a persistent viral load >10^4^ copies/mL in addition to abnormalities in renal function, a presumptive diagnosis of PVAN is made; biopsy is then performed in order to make a definitive diagnosis and stage the nephropathy. Monitoring of BKV DNAemia is therefore performed in order to evaluate the response to the reduction in immune suppression, whereas biopsy is performed to evaluate the course of histological damage [49].

From the laboratory point of view, some technical factors should be taken into account. First, monitoring of BKV-DNA viremia should be performed on the same type of specimen and with the same molecular assay for reliable monitoring over time. Quantitative evaluation of viremia is superior to urine cytology, particularly in terms of sensitivity and positive predictive value. Molecular assays should target conserved genome sequences in order to detect all genotypes and subtypes; moreover, short amplicons (less than 150 base pairs) should be used to avoid subquantification.

Considering BKV DNA quantification, as significant interassay variability precludes the appropriate use of thresholds for the management of BKV infection in transplantation, the 1st WHO International Standard for BKV (primary standard) was introduced in 2016 [50]. However, subsequent studies have evidenced that the WHO standard contains subpopulations of viruses with mutations in the T region that may determine molecular result variations depending on the region of the standard targeted [51]. Therefore, the need to develop commutable international standards for BKV-DNA loads (plasma, whole blood, urine, and tissue) based on defined molecular sequences persists. Another factor to be considered is the clinical importance attributed to a negative assay. On the other hand, it should be taken into account that viral replication does not always mean nephropathy, and cases of high-level and persistent viral replication have been reported in the absence of kidney abnormalities in transplant recipients [52].

No specific antiviral treatment or prophylaxis is currently available for BKV-associated disease. Indeed, the clinical approach commonly consists of a gradual reduction in immunosuppression, guided by evaluation of BKV viremia. However, a long-term reduction in immunosuppression determines an increase in the incidence of chronic rejection. The potential activity of some drugs, including cidofovir and fluoroquinolones, has been evaluated but with no consistent data on efficacy. Despite the lack of specific antivirals, some agents effective on CMV have also been used in association with immunosuppression down-modulation. Unfortunately, most of these studies consist of uncontrolled retrospective observational studies [53]. Another potential treatment consists of the intravenous infusion of specific IgG with the aim of lowering BKV replication [54]. A recent first-in-human study has been conducted to evaluate a novel human IgG1 monoclonal antibody with promising results in terms of the treatment and prevention of BKV disease [55]; at the moment, further data are required to support these results.

As previously mentioned, most cases of PVAN are associated with BKV; however, concurrent JCV-DNAemia has been reported and correlated with a poor graft outcome in kidney transplant recipients with nephropathy. In a study by Zhang et al. [29], concomitant positivity for JCV viremia was evaluated in a population of 140 kidney transplant patients and was found in 18 individuals. In this study, the graft loss rate in the JCV-DNAemia positive group was significantly higher than in the negative group. The authors concluded that JCV-DNAemia was an independent risk factor for graft survival, with an odds ratio of 4.808.

Given the role of immune suppression in the pathogenesis of PVAN, as well as the potential therapeutic strategies, BKV-specific immunity also plays a relevant role in the diagnostic pathway of nephropathy, including both the humoral and cellular arms. As regards the humoral response, standardized and validated serological assays in the transplant setting are lacking, as well as no distinction between serotypes being available (i.e., potentially useful for risk stratification, allocation, and personalized treatment). A clear role for humoral evaluation in the management of kidney transplant patients is not yet defined. However, some studies have evidenced that in seropositive donors, the risks of viremia and nephropathy are higher—particularly in the presence of high levels of IgG with low or undetectable levels in the recipient. Similarly, seropositive recipients before transplantation are not protected against viral replication and PVAN, although a certain level of neutralizing antibody against the donor-specific serotype is related to a lower number of post-transplantation episodes of BKV DNAemia [56,57,58,59,60,61]. Some unmet needs persist in terms of the evaluation of humoral immunity, including the availability of standardized and validated serological assays in the transplant setting and the lack of distinction between serotypes, which must be resolved to achieve risk stratification, organ allocation, and tailored treatment. Considering cellular immunity, this seems to play a role in the course of viral replication. Healthy seropositive subjects present CD4+ and CD8+ T cells that are specific for Tag and VP1, and a reduction in immune suppression is associated with the development and reconstitution of cell-mediated immunity and the clearance of BKV DNAemia [62,63]. Nevertheless, cell-mediated immunity evaluation is difficult to perform, given the differences between methods, the antigens used for stimulation, operating features, timepoints of evaluation, and other factors. Also, in this case, some unmet needs persist including the lack of standardized and clinically validated assays to guide immune suppression and the application of adoptive transfer of virus-specific T cells. In a small study performed by our group using an internally developed ELISPOT assay with an antigenic stimulus obtained from a peptide mix of sequences of VP1 and LargeT antigen, less than 10% of 149 renal transplant patients were responders in the first year following transplantation, with no case of viral reactivation (positivity to viremia and/or viruria) in responders [64]. The potential role of cellular immunity in this context is also evidenced by promising studies on the use of T-cell adoptive immunotherapy with virus-specific T cells; recently, a phase 1 study on kidney transplant patients with PVAN reported preliminary results supporting this treatment as adjunctive therapy for BKV infection post-transplantation; however, larger studies are needed to confirm these data [63].

Re-transplantation may represent an option in selected cases of PVAN. Previously published data reported a three-year survival rate higher than 96%, with an overall rate of recurrence of less than 3% and a rate of graft loss of less than 1%, according to the Review Organ Procurement & Transplantation Network (OPTN) [65]. Among the virological markers predictive of successful re-transplantation, a reduction in more than 2log10 copies of BKV DNA per mL in comparison to the peak value following the immune suppression reduction should be considered, as well as persistent viral clearance before re-transplantation [66]. Several retrospective studies have evaluated the outcome of re-transplantation and concluded that it can be considered acceptable, although the risk of graft loss tends to be higher in comparison to a second kidney transplant in the absence of PVAN.

## 4. Polyomavirus-Associated Nephropathy in Native Kidney from Other Solid-Organ Transplant Recipients

PVAN has been reported in the native kidney of other solid-organ transplant recipients, including liver, lung, heart, and pancreas [67,68,69,70,71,72,73,74,75,76]. In these contexts, BKV-associated nephropathy should be considered in the differential diagnosis based on the occurrence of a recent onset of renal insufficiency. Renal dysfunction and nephropathy are very rare in this context, usually published as case reports. In a systematic review on the incidence and impact of BKV replication in solid-organ transplantation other than the kidney, including almost 100 studies including lung, heart, liver, and pancreas transplants, 17 cases of nephropathy were identified, with the majority of BKV-associated nephropathy cases in heart transplant patients. In these patients, mean creatinine levels were usually higher in subjects with viremia or viruria than in those with no BKV replication. BKV viremia is not routinely monitored in other solid-organ transplantation; when a diagnosis of PVAN is made, viremia usually ranges between 10^4^ and 10^6^ copies/mL. In these cases, a reduction in immune suppression is indicated [21,72,73]. A few cases of BKV nephropathy have also been reported in the native kidneys of AIDS patients, closely related to deep immune suppression [77].

## 5. BK Polyomavirus in Hematopoietic Stem Cell Transplantation

BKV reactivation in HSCT is quite common. Indeed, in patients receiving allogeneic transplantation, uroepithelial injury and the lack of immune reconstitution favor BKV reactivation [78,79]. Viruria and viremia have been reported during follow-up in up to 47–94% and 23–53% of HSCT recipients, respectively [80]. Reactivation may be associated with hemorrhagic cystitis (HC) with an incidence rate of 6% to 29%, particularly in the first year following transplantation, with approximately 15% of cases of HC being grade III/IV [81,82]. BKV-associated HC remains a major complication in HSCT, thus accounting for increased morbidity, prolonged hospitalization, and great health economic burden. Hemorrhagic cystitis post-HSCT presents with dysuria, urgency, and lower abdominal pain in the presence of grade II–IV hematuria [83]. Adenovirus and cytomegalovirus are among the non-infectious and infectious causes of HC in HSCT [82]. Considering virological markers of BKV replication in HSCT, viruria higher than 10^10^ copies/die (or 10^7^ copies/mL) for approximately 30 days (range, 2–118) is considered predictive of post-HSCT HC onset [78], whereas levels of viremia higher than 10^4^ copies/mL for approximately 10 days are considered as related to a high risk of occurrence of post-engraftment HC [84]. Diagnosis of BKV-associated HC in this context is usually performed on the basis of clinical presentation, exclusion of other causes, and evaluation of markers of viral replication (viruria and/or viremia). Routine monitoring of BKV DNA (viruria or viremia) in HSCT is not performed. Therapeutic approaches include immunosuppression modulation and antiviral agents; however, their efficacy is very limited [85,86,87].

Considering hematuria and/or interstitial nephritis in HSCT, very few studies are available. In a study by O’Donnell and colleagues [86] on 124 HSCT recipients, a significant correlation between BKV viruria and hematuria was found, as well as between viremia and serum creatinine increase. In this series, among eight patients in the surveillance cohort, two subjects with persistently positive viremia developed biopsy-proven nephropathy requiring hemodialysis.

## 6. Discussion and Conclusions

In this review, we have summarized various critical aspects of polyomavirus infection in kidney transplant patients, including viral features, risk factors, monitoring strategies, modes of treatment, and outcomes. Despite infection and reactivation being quite common in both immunocompetent and immunocompromised subjects, PVAN was not clearly defined for many years. Only some years ago was it recognized as a specific disease in renal transplant patients, with a need for management strategies for prevention and treatment. Considering the biological behavior of BKV, although the oral route is usually considered the most probable entry route of the virus, this has not been definitely established—nor has the dissemination route of the virus or the mode of transmission. Persistence in the urinary tract is suggested by some widely reported observations, including virus detection in urine from healthy individuals as well as the fact that the main pathologies encountered in immunosuppressed patients are nephropathy and hemorrhagic cystitis. Nevertheless, a huge amount of evidence indicates a wide spectrum of tissue and cell tropisms, including salivary gland cells [88], peripheral blood leukocytes [89], pancreatic cells [90], and endothelial cells [91]. Overall, these findings underline that, although BKV may replicate and reactivate in the urinary tract, many sites of persistence may be recognized. Productive infection and replication of BKV have been widely studied in cell cultures from renal tubule epithelial cells [92]. A role in determining this preferential site for productive infection could be played by the innate immune response against BKV infection, in terms of the activation of IFN signaling and cytokine production. This was accurately studied by An and colleagues [93]. Another interesting context in which potential data on BKV infection/replication and viral behavior could be studied is in lung transplant patients developing nephropathy. In a recent study on 878 lung transplant patients, 6% of recipients developed BKV-associated nephropathy within a period of approximately 30 months [76]. These data evidenced a relatively more frequent incidence of renal damage in these patients in comparison to what was previously expected, thus suggesting a potential for BKV monitoring in these patients, as well as the intriguing suggestion derived from the site of viral entry.

Despite the well-acknowledged etiological role of BKV in the development of PVAN in kidney transplantation recipients, many details of viral behavior including replication, maturation, assembly, and virion release from host cells have not been fully elucidated. Among these, it has been evidenced that BKV DNA may occasionally integrate into the host genome, mainly in patients with BKV-related neoplasms (e.g., urothelial carcinoma); all of the chromosomes are involved, with the exception of the Y chromosome.

However, it is likely that BKV integration alone does not directly induce malignant transformation, and findings from some studies [86] underline the high complexity of BKV-associated cellular transformation and the need to further investigate the mechanisms that determine viral integration and contribute to cancer development [94,95,96].

Although damage to the native kidney has also been described in other solid-organ transplant patients, these cases are quite limited; it could be hypothesized that a specific immunological context characterizes patients with renal diseases leading to transplantation. Further studies are needed on larger series and with the use of clearly defined methods for evaluating viral behavior and immunological status.

In addition to renal transplantation, BK polyomavirus has been associated with the development of hemorrhagic cystitis and hematuria in blood stem cell transplant recipients, whereas the association between BKV and interstitial nephritis in this context is controversial. Few cases have been reported in the literature, also in the absence of hemorrhagic cystitis [97].

Given the limited number of cases reported in the literature, a definitive conclusion cannot be made, in that it could be a co-occurring event or there could be a causative relation, considering the potential role likely to be played by the particular immunological status of bone marrow transplant recipients.

The relevant role played by immunological context in the development of polyomavirus-associated diseases is well acknowledged, particularly for cellular immunity. Nevertheless, whereas methods for evaluating humoral immunity against more common viruses (e.g., cytomegalovirus, Epstein–Barr virus, adenovirus) threatening outcomes in transplant populations are widely consolidated, studies on cellular immunity are much more limited, being mainly available only at research laboratories (e.g., ELISPOT and flow cytometry on B and T cells), and very little information is available for polyomaviruses, in both kidney and stem cell transplant patients. More evidence is available from studies on the cellular immune response to cytomegalovirus, with Interferon-gamma-releasing assays being developed and some being standardized [98]. The availability of standardized assays may allow for studies on larger populations, thus potentially illuminating some aspects of viral pathogenesis. However, it has to be underlined that the often very low mononuclear cell count in stem cell transplant patients may limit the amount of achievable data in this context, in addition to the fact that a completely new immune system develops post-transplant in this particular cohort. The role of cellular immunity in the onset of virus-associated disease in bone marrow transplantation is also evidenced by the clinical and therapeutic potential of adoptively transferred virus-specific T cells to prevent or treat viral infections and related complications in solid-organ and HSCT recipients [99]. In terms of potential modalities of treatment, in addition to adoptive T-cell-based treatment, preliminary results have also been reported on the use of specific Igs; however, given the limited amount of data on this topic, further studies are required. It should also be considered that, apart from a specific viral behavior, differences between the reactivation of the primarily infecting viral strain and a new primary infection with a virus strain transmitted by a donor kidney could also impact replication capability, immunological response, and therefore transplant outcome. Other confounding factors could impact the occurrence of viral reactivation and immunological status, for example, the availability of data on the overall risk of infection (such as those obtained by means of non-pathogen-specific immunological assays); also, including the risk for bacterial and other viral infections could allow for more precise definition of the clinical context of each patient.

Considering monitoring for viral replication, although viruria may represent an early predictor of viral reactivation and precede viremia, given the fact that asymptomatic viruria may also occur without the subsequent occurrence of viremia, a more cost-effective approach is to only evaluate the occurrence of viremia with a monthly frequency in the period of highest risk (i.e., first year). In this context, local practice can be supported by an evaluation of the cost–benefit ratio in terms of the costs of monitoring, laboratory analyses, the implemented immunosuppression strategy and its monitoring, a prolonged hospital stay period, and the incidence of events such as infectious and rejection complications. Similarly, the potential costs related to the need for return in hemodialysis due to graft loss after PVAN should also be considered. In a large systematic review by Eder and colleagues [36], risk factors for the development of BKV-associated complications were evaluated. It should be noted that the high number of cases included in this meta-analysis (i.e., 197,029) lends support to the conclusions, although only 23% of these had a prospective design and 29 were considered at a high risk of bias, such as those considering different immunosuppressive protocols. Another aspect to consider is the need for affordable BK virological monitoring in the overall management of renal transplant patients. In this context, focusing on the use of widely used and standardized molecular methods relying on the availability of the WHO standard is crucial for clinical management and the reproducibility of collected data in clinical studies.

In conclusion, PVAN still represents a potential threat in renal transplantation. Its pathogenesis mainly results from immunosuppression with a combination of virus-induced graft damage and lack of adequate host immune response. Diagnosis is usually performed by histopathological evaluation, taking into account the potential for false negative results. The monitoring of viral replication is routinely recommended and may be useful for a presumptive diagnosis of kidney damage and the need for preemptive modulation of immunosuppression. Knowledge of viral biology could be useful for an accurate evaluation of virological diagnosis and monitoring. The limited therapeutic armamentarium of antiviral agents warrants the need for further studies on larger populations and exploration of new biomolecules and/or potential targets.

## Figures and Tables

**Table 1:** Polyomaviruses of interest in humans. Two other primate viruses have also been reported in humans. BK and JC, the initials of the patients in which the viruses were first isolated; KI, Karolinska Institute; WU, Washington University; PyV, polyomavirus; HPyV, human polyomavirus; IS, immunosuppression; PML, progressive multifocal leukoencephalopathy; IC, immune competence; TS, trichodysplasia spinulosa. The reference of the first description is reported in the table.

Virus	Human Pathologies	Immune Status	First Description
BK	Nephropathy, hemorrhagic cystitis	IS	1971 urine, kidney transplant pt [2]
JC	PML	IS	1971 postmortem brain, PML [3]
KI	Mild infection of respiratory tract?	IC + IS	2007 [4]
WU	Mild infection of respiratory tract?	IC + IS	2007 [5]
Merkel cell PyV	Merkel cell carcinoma	IC + IS	2008 [6]
HPyV6	Epithelial proliferation, itching, dyskeratotic dermatitis (sporadic), keratoacanthoma?	IS	2010 [7]
HPyV7	Epithelial proliferation, itching, dyskeratotic dermatitis (sporadic), thymoma?	IS	2010 [7]
Trichodysplasia spinulosa PyV	TS	IS	2010 [8]
HPyV9	Hyperkeratotic papules and plaques (sporadic)	IS	2011 [9]
HPyV10 and variants MWPyV, MXPyV	-	-	2012 [10]
Saint-Louis PyV	Infant diarrhea?	-	2013 [11]
HPyV12	-	-	2013 [12]
New Jersey PyV13	Myositis, cutaneous necrosis, vasculitis (1 case)	IS	2014 [13]
Lyon IARC PyV	Infant diarrhea?	-	2017 [14]
Quebec PyV	-	-	2019 [15]

**Table 2:** Monitoring of BK polyomavirus in transplant recipients, according to the Second International Consensus Guidelines on the Management of BK Polyomavirus in Kidney Transplantation [38].

Kidney Transplantation	Bkv-Dna Quantitation On Plasma Monthly up to month 9. Every three months from month 10 to month 24. Monthly for three months in the occurrence of graft dysfunction or biopsy performed for surveillance or upon clinical suspicion (rejection or nephropathy). In cases of persistent viremia (>10^3–4^ copies/mL), confirmation within 2–3 weeks and monitoring every 2–4 weeks.
Kidney Combined Transplantation	Bkv-Dna Quantitation On Plasma Extension up to month 36.
Other Solid-Organ Transplantation	Routine evaluation not indicated, consider in the occurrence of renal dysfunction.

## Data Availability

Not applicable.

## Abbreviations

The following abbreviations are used in this manuscript:

PVAN	Polyomavirus-associated nephropathy
HSCT	Hematopoietic stem cell transplantation
HC	Hemorrhagic cystitis
